# Validation of the prognostic model “oncologiq palliative” for head and neck cancer patients

**DOI:** 10.1007/s00405-025-09462-1

**Published:** 2025-05-24

**Authors:** B. N. van den Besselaar, D. van Klaveren, D. Berzenji, A. Hoesseini, J. C. Jansen, J. A. Hardillo, R. J. Baatenburg de Jong, M. P. J. Offerman, A. Sewnaik

**Affiliations:** 1 Department of Otorhinolaryngology and Head and Neck Surgery, Erasmus MC Cancer Institute, University Medical Center Rotterdam, Rotterdam, The Netherlands; 2 Department of Public Health, Erasmus University Medical Centre, Rotterdam, The Netherlands; 3 Department of Otolaryngology and Head and Neck Surgery, Leiden University Medical Center, Leiden, The Netherlands

**Keywords:** Head and neck Cancer, Personalized prognostic counselling, Prognostic modelling, Palliative care, Proactive care planning, Validation study

## Abstract

**Purpose:**

Patients diagnosed with incurable head and neck squamous cell carcinoma (HNSCC) have a poor prognosis, with a median survival of approximately five months. Physicians often struggle to predict prognosis accurately and tend to overestimate survival. Timely sharing of validated accurate individual information on life expectancy could aid in facilitating better patient counseling. However, this knowledge is lacking. Therefore, the aim of this study is to conduct temporal and external validation of the prognostic model OncologIQ Palliative to assess its accuracy and generalizability.

**Methods:**

The validation procedure involved temporal assessment in a retrospective cohort of 355 palliative HNSCC patients from Erasmus MC (2017–2020), followed by external validation in a retrospective cohort of 44 patients from Leiden University Medical Center (2019–2021).The discriminative ability of OncologIQ Palliative was assessed using the C-index and calibration was evaluated through graphical assessment, intercept, and slope.

**Results:**

The temporal cohort had a median follow-up of 115 days, and the external cohort 143 days. The model showed moderate discriminative ability in temporal validation (C-index 0.66) and better discrimination in external validation (C-index 0.71). Reasonable agreement was observed between predicted and observed 6-month and 1-year survival rates, with some deviations from the perfect calibration line.

**Conclusion:**

The validation methods conducted in this study underscored the reliability of OncologIQ Palliative. They showed adequate calibration and discrimination in both validation procedures, thereby facilitating the provision of more accurate prognostic counselling for head and neck cancer patients in the palliative phase.

## Introduction

Overall, patients with head and neck squamous cell cancer (HNSCC) have a poor prognosis, with almost half of patients dying as a result of their disease [1]. This poor outcome is due to high recurrence rates (up to 50%), as well as other factors such as a higher comorbidity burden, lifestyle factors and socioeconomic status in the HNSCC population [2, 3]. Consequently, a large group of patients will enter the palliative phase at some point [4]. This phase starts when cure is no longer possible or when patients refuse curative treatment. During this phase, patients experience a wide range of specific symptoms like dyspnoea, pain, speech, psychosocial problems and changes in appearance [5–10]. In general, survival is short with a median survival of approximately 5 months, although it can vary from a matter of days to even years [4, 11–13]. Adequate palliative care can aid to maintain quality of life of patients and their loved ones for as long as attainable and help them make their remaining life as comfortable as possible [14]. 

Various studies uncovered the need of patients with incurable cancer to receive more prognostic information [15, 16]. This desire to acquire more quantitative information in case of an unfavourable prognosis has also been expressed by HNSCC patients [17]. Furthermore, van der Velden observed that individuals prefer numerical over word-based estimates [18]. The integration of quantitative estimations by physicians during prognosis discussions may not only enhance patients’ understanding of prognosis but also facilitate more informed treatment decision-making [19]. This effect even persists when a physician explicitly states that the prognosis is unknown, as opposed to providing no information at all [18]. However, physicians often refrain from providing individualized prognoses, possibly because they tend to overestimate patient survival [20, 21]. Enhancing the accuracy of survival prediction supports physicians to engage in informed prognostic discussions in palliative care. This, in turn, could empower patients and their caregivers to make well-considered end-of-life decisions and optimize palliative care planning.

To cater to these wishes and preferences expressed by HNSCC patients to receive more prognostic information, we developed the prognostic model OncologIQ Palliative [13]. This model integrates seven predictors, combining tumour-specific factors (TNM-classification) with patient-specific variables (prior HNSCC, WHO-performance status, reason for entering the palliative phase and weight loss in the 6 months before diagnosis), to calculate the individual overall survival (OS) probability [13]. Utilizing this model in counselling allows clinicians to improve the accuracy of individual survival predictions for patients in the palliative phase, contributing to more informed prognostic discussions.

Despite the development of numerous prognostic models, successful implementation into clinical practice remains limited. This may be attributed to the fact that the impact of using such models and how this information should be shared with patients is unknown. Another possible reason is the lack of adequate validation of many prognostic models [22], partially due to the challenge of obtaining independent datasets of patients with similar variables tot those in the development set. Yet, it is crucial to validate and assess the performance of prognostic models in different cohorts. In prognostic modelling, a typical problem is overfitting, which can result in predictions that are either excessively low or high. While the predictive accuracy might appear satisfactory when tested within the development dataset, the model’s performance could be poor when assessed with a new dataset [23, 24]. 

Therefore, it is important to perform external validation before using a prognostic model in clinical practice [22, 25]. We aimed to conduct both temporal and external validation of OncologIQ Palliative to assess the model’s accuracy and generalizability to other populations.

## Methods

### Datasets

The data utilized in this study were retrieved from the electronic health records (EHR) of the Erasmus MC and the Leiden University Medical Centre (LUMC). The temporal set encompassed patients from the Erasmus MC, while the external dataset was derived from the LUMC.

All patients who were diagnosed at the Erasmus MC Cancer Institute with incurable HNSCC or who refused curative treatment for HNSCC between January 1st, 2017 and December 31st, 2020 were included in the temporal set. Patients with incurable HNSCC or who refused curative treatment for HNSCC from the LUMC between January 1st, 2019 and November 30th, 2021 were gathered from the EHR to form the external dataset.

In both cohorts, the baseline characteristics were from the time of the palliative diagnosis. The final day of follow-up for a patient was specified as the last date that the patient was confirmed to be alive or the date of death. This was last updated on the 31st of March 2023, by consulting the EHR.

### Patient inclusion

Data on all patients diagnosed with incurable HNSCC or those who refused curative treatment for HNSCC were collected from both medical centres. These datasets were reviewed and patients with the following locations of HNSCC were selected: glottic larynx, supraglottic larynx, hypopharynx, oropharynx, oral cavity, nasopharynx and unknown. Patients were then included in either the temporal or external dataset. In total 355 patients were entered in the temporal dataset and the external dataset encompassed 44 patients. Patients were considered to have incurable HNSCC either based on tumour-related factors (such as distant metastasis, inoperability with no viable curative treatment alternatives) or patient-related factors (such as severe comorbidity). In the temporal dataset, the palliative diagnosis of 282 patients was attributed to tumour and patient factors, while an additional 73 patients declined treatment. In the external cohort, the palliative diagnosis for 29 patients was due to tumour and patient factors, and 13 patients refused treatment, while information was missing for 2 patients.

### Variables

All tumor- and patient specific data were scored at the date of diagnosis of the palliative tumour. The clinical TNM was scored according to the 8th American joint committee on cancer (AJCC) edition of the TNM classification [26]. Previous HNSCC was defined as the total number of HNSCC’s before palliative diagnosis. The weight loss was defined as weight loss in kilograms in the six months prior to palliative diagnosis. WHO-performance status, was scored according to the classification by Oken et al. [27]

### Statistical analysis

All statistical analyses were conducted using IBM^©^ SPSS^©^ Version 28 and R (version 4.2.3). Descriptive statistics were used to analyse demographic variables and tumour characteristics of both cohorts. To handle the missing data multiple imputation in R was used [28]. Follow-up time was calculated for each patient from the time of diagnosis to either the date of death or March 31st 2023, whichever followed first.

### Model performance indicators

Performance of OncologIQ Palliative was assessed through calibration and discrimination. The calibration plots determine whether predictions align with the observed outcomes. Calibration was examined for 6-months and 1-year survival rates. The observed survival frequency was plotted against the predicted survival probability according to the prognostic model. A calibration intercept (a) of less than zero indicates an overestimation of survival, while an intercept above zero represents an underestimation of survival. A slope (b) of less than one signifies weaker associations between predictors and survival, whereas a slope of more than one indicates a stronger association. Discrimination expresses the ability of a prognostic model to differentiate between patients who survive and those who die. The commonly applied Concordance index (C-index) quantifies this discrimination by estimating the probability that, in a randomly selected pair of patients, the one with the worse predicted outcome is the patient who dies first. The C-index has a value between 0.5 and 1.0, where 0.5 implies that the model is not better than chance and 1 indicates perfect discrimination. Usually a C-statistic below 0.6 can be considered as poor, over 0.6 as moderate, over 0.7 as good and over 0.8 as strong [29]. 

### Temporal & external validation

We assessed discrimination and calibration of OncologIQ Palliative in new patients from the same centre (EMC; temporal validation) and in new patients from a different centre (LUMC; external validation). This was conducted to confirm accuracy across not only a novel local population but also a distinct population. During the development, validation and reporting of the model we utilized the guidelines provided by the Transparent Reporting of a multivariable prediction model for Individual Prognosis or Diagnosis (TRIPOD) statement [30]. 

## Results

Overall, the temporal validation cohort and the external validation demonstrated a considerable degree of similarity (Table 1). The diagnostic timeframe of the external cohort (LUMC) spans from 2019 to 2021 and the temporal cohort (Erasmus MC) from 2017 to 2020. The cohorts had median follow-up times of 143 days and 115 days, respectively. 97.5% of patients from the Erasmus MC cohort and 90.9% from the LUMC cohort had passed away before the end of last follow-up. Some predictor distributions differed between both cohorts. Most patients in the Erasmus MC had an oral cavity tumour, while this tumour location represents only 18.2% of the LUMC population. In addition, 63.1% of patients in the Erasmus MC had one or more tumours before their palliative diagnosis versus 21.5% in the LUMC cohort.

**Table 1:** Demographic and follow-up data of the different patient cohorts. EMC, Erasmus medical center; LUMC, Leiden university medical center; N, number of patients; N(%), percentage of total number of patients; IQR, interquartile range; SD, standard deviation

Variable	Temporal cohort (EMC) *N* (%)/Median (IQR)/Mean (SD)	External cohort (LUMC) *N* (%)/Median (IQR)/Mean (SD)	*P*-value
Total	355	44	
Age	69.2 (11.5)	67.2 (10.1)	0.272
Sex			0.910
Female	110 (31.0%)	14 (31.8%)	
Male	245 (69.0%)	30 (68.2%)	
Tumour location			0.232
Oropharynx	102 (28.7%)	18 (40.9%)	
Oral cavity	116 (32.7%)	8 (18.2%)	
Hypopharynx	56 (15.8%)	6 (13.6%)	
Nasopharynx	14 (3.9%)	3 (6.8%)	
Supraglottic larynx	26 (7.3%)	5 (11.4%)	
Glottic larynx	25 (7.0%)	1 (2.3%)	
Unknown primary	16 (4.5%)	3 (6.8%)	
T classification			**0.009**
T0	111 (31.3%)	3 (6.8%)	
T1	8 (2.3%)	2 (4.5%)	
T2	38 (10.7%)	9 (20.5%)	
T3	49 (13.8%)	6 (13.6%)	
T4	137 (38.6%)	22 (50.0%)	
Missing	12 (3.4%)	2 (4.5%)	
N classification			0.132
N0	163 (45.9%)	13 (29.5%)	
N1	30 (8.5%)	6 (13.6%)	
N2	97 (27.3%)	17 (38.6%)	
N3	59 (16.6%)	6 (13.6%)	
Missing	6 (1.7%)	2 (4.5%)	
M classification			0.685
M0	229 (64.5%)	27 (61.4%)	
M1	118 (33.2%)	12 (27.3%)	
Missing	8 (2.3%)	5 (11.4%)	
Previous HNC			**< 0.001**
0	131 (36.9%)	35 (79.5%)	
1	126 (35.5%)	4 (9.1%)	
2	68 (19.2%)	2 (4.5%)	
≥3	30 (8.5%)	3 (6.8%)	
WHO-performance status			0.720
0	45 (12.7%)	6 (13.6%)	
1	108 (30.4%)	11 (25.0%)	
2	98 (27.6%)	7 (15.9%)	
3 + 4	85 (23.9%)	7 (15.9%)	
Missing	19 (5.4%)	13 (29.5%)	
Weight loss in last 6 months	4.0 (8)	3.0 (10)	0.846
Missing	26 (7.3%)	7 (15.9%)	
Reason palliative			0.122
Incurable	282 (79.4%)	29 (65.9%)	
Choice patient	73 (20.6%)	13 (29.5%)	
Missing	-	2 (4.5%)	
FU-time days	115 (157)	143 (164)	0.089
Status at last follow-up			**0.044**
Dead	346 (97.5%)	40 (90.9%)	
Alive	9 (2.5%)	4 (9.1%)	

### Temporal validation

**Fig. 1:**
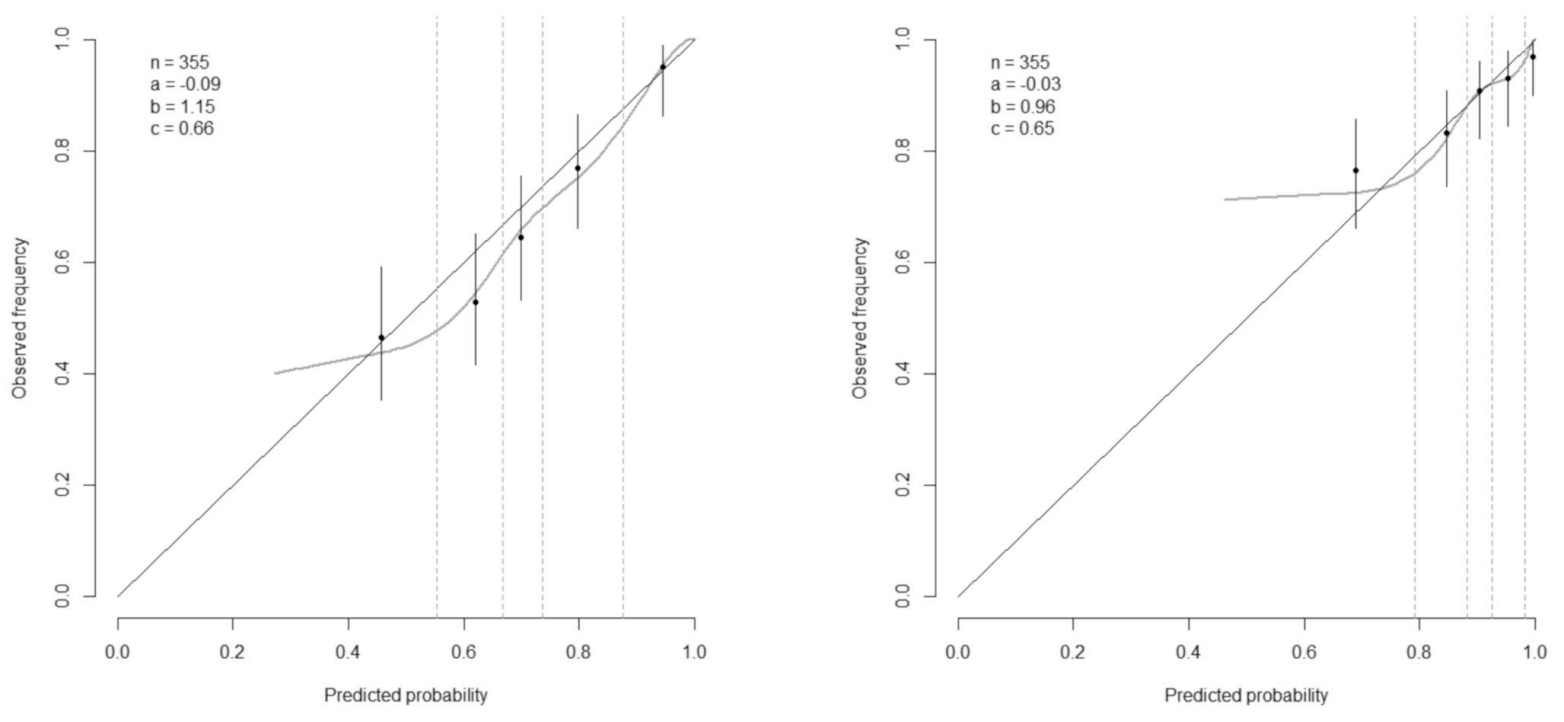
Temporal calibration plots of predicted survival probabilities and observed deaths in five quintiles at 6 months (left) and 1 year (right) after diagnosis. n: patients, **a**: intercept, **b**: slope, **c**: c-statistic

Predictions and observed outcomes for 6-months and 1-year survival showed reasonable agreement (Fig. 1). The predicted probabilities were slightly higher than the observed frequencies in this cohort, with a calibration intercept of -0.09 at six months and − 0.03 at one year. The calibration slope was 1.15 (95% CI, 0.90–1.40) at six months and 0.96 (95% CI, 0.74–1.18) at one year. The discriminative ability was moderate, with a C-statistic of 0.66 (95% CI, 0.62–0.69) at six months and 0.65 (95% CI, 0.61–0.68) at one year, but in line with the previously reported discrimination of the original palliative model in the development data (0.66) [13]. 

### External validation

**Fig. 2:**
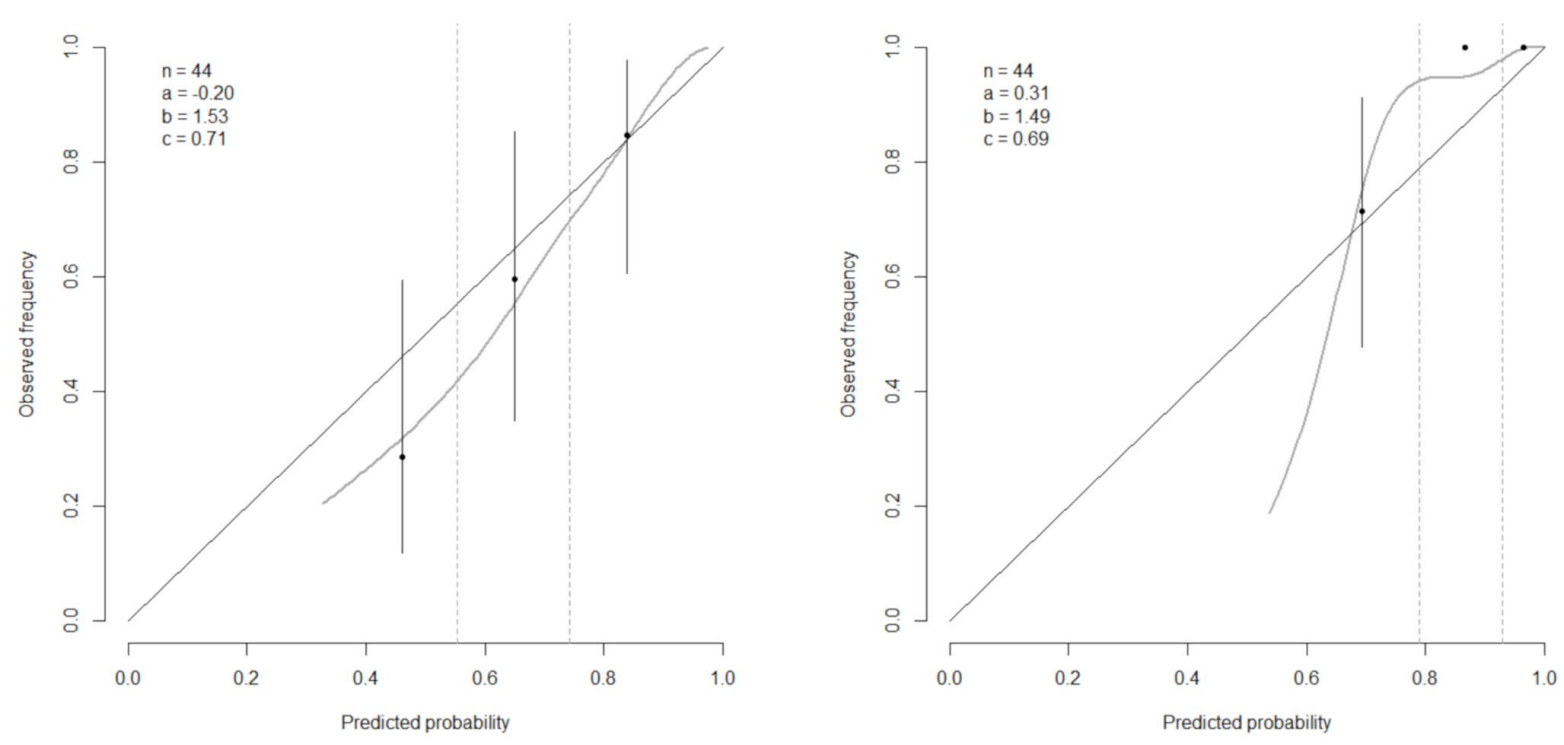
External calibration plots of predicted survival probabilities and observed deaths in three risk categories at 6 months (left) and 1 year (right) after diagnosis. n: patients, **a**: intercept, **b**: slope, **c**: c-statistic

Predictions and observed outcomes for 6-months and 1-year survival displayed decent agreement in the external cohort of LUMC patients (Fig. 2). However, predicted probabilities for OS in the external population were higher than the observed frequencies at 6-months, with an intercept of -0.2, but lower at 1-year, with an intercept of 0.31. The association between predictors and survival was stronger in the external validation cohort (calibration slopes 1.53 (95% CI, 0.42–2.64) and 1.49 (95% CI, 0.58–2.41)). This was also reflected by a better discriminative performance, with C-statistics of 0.71 (95% CI, 0.59–0.82) at 6 months and 0.69 (95% CI, 0.59–0.79) at 1 year).

## Discussion

This study describes the temporal and external validation of the prognostic model OncologIQ Palliative. The model was developed with 656 consecutive HNSCC patients in the palliative phase and predicts a personalized survival probability based on readily available prognostic factors. Discrimination in the temporal validation set exhibited moderate performance, with a C-index of 0.66 (95% CI, 0.62–0.69). Better discrimination was noted in the external validation set (C-index 0.71; 95% CI, 0.59–0.82). There was reasonable agreement between predicted and observed 6-months and 1-year survival rates, despite some deviations from the ideal calibration line.

Given that the majority of prognostic models lack (adequate) validation [22, 25, 31–33], our study stands out. Despite the challenge of obtaining independent datasets with similar variables available to those in the original development set, we successfully validated the prognostic model OncologIQ Palliative using new datasets from the Erasmus MC and the LUMC. Initially, these datasets appear quite similar, but closer examination revealed some notable differences in baseline characteristics. For instance, the LUMC dataset showed a reasonably lower percentage of T0 tumours (6.8% vs. 31.3%) and a higher proportion of patients with no previous history of HNSCC tumours (79.5% vs. 36.9%). Despite these discrepancies, the good results observed in the external validation procedure are noteworthy, particularly given the higher C-index in the external cohort compared to the development and temporal cohorts (0.71 vs. 0.66 and 0.66). Several factors may contribute to this difference. One possibility is that certain variables used in the model might have varying impacts on survival across different patient populations. In the external cohort, there seems to be a stronger association between specific variables and survival, leading to better model performance. Additionally, it is possible that the external cohort includes more patients with extreme risk profiles, facilitating easier discrimination between individuals [25]. Notably, despite the higher C-index, a lower calibration performance was observed during external validation. This is likely to be attributed to the smaller sample size (*n* = 44), which could introduce more random fluctuations.

Previous research amongst advanced cancer patients has demonstrated fair efficiency in predicting mortality over a one-year period [33], while other studies involving diverse types and stages of cancer patients have shown that machine learning can effectively predict mortality [34, 35]. These studies reported high accuracy levels that matched or surpassed the performance of our current model. However, the relevance of these findings may be limited in the context of HNC patients. The median survival in the palliative HNC population is generally significantly shorter with a median of five months [4, 11, 13], which raises questions about the relevance of a model that predicts one-year mortality. Furthermore, the specific impact of the various variables used in machine learning algorithms remains unclear and is not tailored for palliative HNC patients, who represent a distinct population with unique characteristics and circumstances [7–10]. Therefore, it is essential to further explore the support and applicability of our prognostic model among healthcare professionals in routine clinical practice.

Although this study demonstrated moderate discriminative performance, more prognostic factors may need to be introduced to even further improve the performance of this prognostic model. In patients with distant metastasis, it is known that HNSCC patients with limited distant metastatic spread have better survival rates than those with more aggressive spread [12]. By introducing this concept of “oligometastasis” into the prognostic model, and thus subdividing the variable of distant metastasis into “oligometastasis” and “polymetastasis”, more discriminatory power may be achieved. In addition, virus-driven head and neck cancers, such as Human Papillomavirus (HPV) p16 or Epstein-Barr virus, may also impact survival in case of palliative diagnosis. For example, it is known that HPV p16-positive tumours have better survival than HPV p16-negative tumours, and that HPV p16-positive tumours metastasize later in their course than their p16-negative counterparts [12, 36]. It is, however, still ill-defined in literature how virus-status further impacts survival after palliative diagnosis.

Since external validation of a prognostic model is crucial before its implementation in clinical practice, the results of this study provide greater confidence in the clinical implementation of OncologIQ Palliative. However, before proceeding with the implementation of this model as standard of care, it is crucial to evaluate the impact of counselling patients using this model and explore how the information should be shared.

### Strengths and limitations

This study has multiple strengths, including a large temporal cohort size (*n* = 355) and a high number of events in both datasets. The consecutive inclusion of patients eliminates selection bias, combined with the minimal missing data, strengthens the reliability of our results. However, the variable WHO-performance status from the external dataset had a slightly higher percentage of missing values (29.5%). To address this issue, we employed multiple imputation to manage missing values effectively and prevent any potential data loss [37]. Additionally, to our knowledge we are one of the first to temporally and externally validate a prognostic model for palliative patients [22, 25]. However, its applicability outside of the Netherlands may be limited due to potential population differences, considering that our model was developed and validated using datasets comprised of Dutch patients. Therefore, prior to considering its implementation in other countries, additional validation procedures are essential. Nevertheless, upon successful validation, the model holds promise for extrapolation to various other regions.

One limitation of our study was the relatively small group of patients in the external cohort. Nonetheless, ongoing efforts involve further external validation using larger and more diverse datasets. However, some studies even doubt the necessity of external validation [38]. We did not include HPV determination, nor did we categorize within de M1 category. Despite these limitations, our study has the potential to provide more accurate personalized prognostic information for patients with HNSCC in the palliative phase.

### Future perspectives

Following the validation of OncologIQ Palliative, further steps can be taken towards successful integration of the model into clinical practice [39]. We will explore how clinicians can effectively communicate the prognostic information to patients using the model. This includes exploring user needs and preferences for visualising the model as an online dashboard, prioritizing user-friendliness and accessibility. Navigating these topics with patients in the palliative phase presents unique challenges due to their shorter lifespan and specific needs regarding prognostic information. However, we can tailor our approach by building upon our prior experiences with developing OncologIQ for patients in the curative phase [17]. 

We will conduct qualitative research, exploring the impact of using such a model in the palliative phase, and understand needs and communication preferences of both patients and professionals. By actively involving patients and professionals in co-creation, we establish an ongoing cycle between research and care. This iterative process allows us to continuously drive innovation, address any challenge and optimize implementation from the beginning. The results of this qualitative research will support physicians to effectively communicate the outcome of the model, which can contribute to more informed prognostic discussions. This in turn can attribute to more patient centred counselling and facilitate better palliative care planning. Furthermore, it may enable patients and their caregivers to make more well-informed decisions and a better preparation of their end-of-life care.

## Conclusion

This study enabled validation of the previously developed prognostic model, OncologIQ palliative. It demonstrated moderate calibration and discrimination results in both temporal and external validation procedures, underscoring the fair reliability of the model. The model provides more accurate personalized prognostic counselling in the palliative phase. However, before being utilized, it is essential to conduct qualitative research and assess user needs regarding communication and visualization of the model in an online dashboard.

## Declarations

### Presentations

An oral presentation of this article was held at the JOD Congress in Nijmegen, the Netherlands, on May 24, 2024. We received first prize for the best and most relevant presentation. An oral presentation of this article was held at the 7th CEORL-HNS Congress in Dublin, Ireland, on June 18, 2024.
